# Assessment of the Level of Satisfaction with Medical Care of Patients Treated in Osteoporosis Clinics as an Indicator of the Quality of Medical Care

**DOI:** 10.3390/ijerph19127343

**Published:** 2022-06-15

**Authors:** Agnieszka Barańska, Urszula Religioni, Bartłomiej Drop, Magdalena Bogdan, Anna Kłak, Andrzej Warunek, Jolanta Herda, Ewelina Firlej, Piotr Merks

**Affiliations:** 1 Department of Medical Informatics and Statistics with e-Health Lab, Medical University of Lublin, K. Jaczewskiego 5 Street, 20-059 Lublin, Poland; bartlomiej.drop@umlub.pl; 2 School of Public Health, Centre of Postgraduate Medical Education of Warsaw, 01-826 Warsaw, Poland; urszula.religioni@gmail.com; 3 Collegium of Business Administration, Warsaw School of Economics, 02-513 Warsaw, Poland; 4 Department of Social Medicine and Public Health, Medical University of Warsaw, 02-007 Warsaw, Poland; mbogdan@wum.edu.pl; 5 Department of Environmental Hazards Prevention, Allergology and Immunology, Medical University of Warsaw, 02-091 Warsaw, Poland; anna.klak@wum.edu.pl; 6 National Center for Healthcare Quality Monitoring, 30-347 Krakow, Poland; warunek@cmj.org.pl; 7 Department of Public Health, Medical University of Lublin, 20-090 Lublin, Poland; jolantaherda@umlub.pl; 8 Department of Cosmetology and Aesthetic Medicine, Medical University of Lublin, 20-090 Lublin, Poland; ewelinafirlej@umlub.pl; 9 Department of Pharmacology and Clinical Pharmacology, Faculty of Medicine, Collegium Medicum, Cardinal Stefan Wyszyński University, 01-815 Warsaw, Poland; p.merks@uksw.edu.pl

**Keywords:** satisfaction with medical care, osteoporosis, quality of medical care, osteoporosis clinics, medical entities, healthcare

## Abstract

The aims of this research are to assess the level of satisfaction with medical care among patients treated in osteoporosis clinics and to determine the relationship among the frequency of visits to the doctor, the duration of treatment, socio-demographic factors, and patient satisfaction with the medical care they receive. The study was conducted from August 2016 to July 2018 at osteoporosis clinics in eastern Poland. The study participants were 312 patients treated for osteopenia or osteoporosis. The authors utilized two research instruments: the PASAT POZ questionnaire and their own questionnaire. The results indicate that the duration of osteoporosis treatment is a factor that significantly influences the level of satisfaction with medical care: the longer the treatment time, the poorer the assessment of the clinic, and therefore, the lower the patient degree of satisfaction. Our analysis shows that women assess clinics more positively overall. Additionally, the higher the study participants’ age, the lower the general assessment of the clinic. A further analysis showed that respondents in better financial situations and with higher levels of education tended to assess clinics more favorably. The Pareto-Lorenz analysis indicated that the key element in general assessments of specialist clinics is the doctor. It is advisable for health service providers to monitor the quality of health care they are providing and make improvements. Therefore, further research is needed, especially in relation to chronic diseases such as osteoporosis.

## 1. Introduction

Monitoring the quality of medical care in healthcare systems is not a new phenomenon; however, it is still insufficiently researched. Patient satisfaction with care is a measure of the degree of agreement between his/her expectations of care and the perception of what he/she actually receives [1]. One of the most significant issues concerning measuring satisfaction with care is to determine the likely impact of individual expectations on the level of satisfaction with medical services [2,3]. In order to assess the quality of care, the patient needs to compare his/her own experiences and feelings to his/her expectations [4].

Satisfaction is an aspect that can significantly influence the position of a healthcare entity on the medical services market [5]. The primary objective of undertaking healthcare-satisfaction survey in medical institutions is to bring about improvements of the quality of services. As studies have confirmed that low patient satisfaction with non-hospital services causes more frequent hospitalizations [6,7,8], conducting research on patient satisfaction and the associated loyalty of patients can benefit healthcare facilities. Understanding a patient’s needs and requirements in terms of services and overall healthcare provision can serve as a basis in the process of improving the quality of care. The quality of provided medical services translates not only into the trust and safety of patients, but, above all, into positive health outcomes [5,9,10,11].

The issue of assessing the quality of medical services, especially in the context of civilization diseases, is important to many institutions, e.g., healthcare providers and recipients, local governments, managers of medical entities, politicians and taxpayers [12,13]. Based on epidemiological data and cost analyses related to its prevention, diagnosis and treatment, osteoporosis, a metabolic bone tissue disease, is considered a social and economic problem [14,15,16]. Osteoporosis is recognized by the World Health Organization as a civilization disease and an epidemic of the 21st century [17,18,19]. It is the most common bone disease in humans, affecting both genders and all races [20]. By analyzing the epidemiology data of osteoporosis, it can be concluded that it is a disease that requires particular attention from health professionals and public health experts [21,22,23]. As care for people and their health represent the essence of any healthcare system, patients’ needs and expectations should be a major focus of medical personnel [13]. Healthcare recipients are the main figures in the process of providing care; they have the right to high-quality medical services and to co-decide on the course of their treatment and nursing process [24]. Patient satisfaction is a multidimensional phenomenon; its assessment makes it possible to analyze discrepancies between what the patient considers to be good medical service and what the healthcare provider considers appropriate.

The aims of this research are to assess the level of satisfaction with medical care among patients treated in osteoporosis clinics and to determine the relationship between the frequency of visits to the doctor, the duration of treatment, socio-demographic factors, and patient satisfaction with medical care.

## 2. Materials and Methods

### 2.1. Study Design

This study was conducted at the osteoporosis clinics in Lublin, Świdnik and Zamość (eastern Poland) in the period from August 2016 to July 2018. The study was voluntary, and each participant signed a consent form and was assured that the study was anonymous. The purpose of the study was explained to the respondents, as well as how they should independently complete questionnaires. One of the researchers was on hand to help in the case of any difficulties in terms of understanding the language in the questionnaire.

### 2.2. Patients

The study participants were 312 patients treated for osteopenia or osteoporosis, including 286 women and 26 men (mean age—63 ± 9, range—45–88 years). The inclusion criteria were as follows:

− Women and men being 45 years old or older;
− Patients treated for osteopenia or osteoporosis for at least one year;
− Granting consent to participate in the study.

Exclusion criteria: lack of consent to participate in the study.

Osteopenia is a decrease in bone mineral density (bone mass is reduced by about 2%). This disease is a signifier of the early stages of osteoporosis. Densitometry is a test that allows the measurement of bone mass and the visualization of the structure of the bone. According to the standards developed by the World Health Organization and the International Society for Clinical Densitometry, a T-score value below −2.5 in postmenopausal women and men over 50 years of age is determinant of the presence of osteoporosis. Osteopenia is characterized by a T-score within the range of −1 to −2.5.

### 2.3. Questionnaires

In order to obtain research material, a diagnostic survey method was used. The authors applied two research instruments:

- The PASAT POZ questionnaire, a tool for measuring patient satisfaction in specialist and primary health care clinics. The tool was developed by the team from the National Center for Healthcare Quality Monitoring in Krakow, Poland. Cronbach’s alpha reliability coefficient is 0.9283. The questionnaire consists of 21 questions, and among the identified and assessed attributes of healthcare are: registration, clinic assessment, medical care, nursing care, other aspects of medical care (information, support), as well as a general assessment. The PASAT tool group is available for a fee. The fee was covered by the project’s funding for the Development of PhD and Young Scientists of the Ministry of Science and Higher Education, the director of which is the lead author of the current publication.
- A questionnaire prepared by the authors, consisting of questions on self-assessments of health, duration of osteoporosis treatment and diagnostic methods used, as well as socio-demographic data.

### 2.4. Ethical Issues

The study was conducted in accordance with the human research principles in the Helsinki Declaration after obtaining the consent of the Bioethics Committee of the Medical University of Lublin, Poland, confirmed by the decision number KE-0254/175/2016.

### 2.5. Statistical Analysis

Continuous variables were reported as means (M) ± standard deviation (SD), median (ME), interquartile range (IQR) and minimum (MIN)–maximum (MAX) range. The Mann Whitney U test was used to ascertain whether there was a statistically significant difference concerning the rank variables between two groups. Analysis of variance (ANOVA) was applied to identify if there was a statistically significant difference concerning ratio variables between more than two groups. In order to examine which groups differed from each other significantly, a Turkey’s multiple comparison test was performed. Spearman’s rho was applied to evaluate whether there were any statistically significant correlations between the rank variables and the ratio variables. The Student’s *t*-test was utilized to examine whether there were any statistical differences in terms of the ratio variables between the two groups. Before parametric tests were applied, assumptions on the normality of distribution were verified by means of the Shapiro-Wilk test. A *p*-value of <0.05 defined the statistical significance of differences. Analyses were performed using the Statistica 11 software, Kraków, Poland.

A Pareto-Lorenz analysis was applied to the results of the study. Such an analysis assumes that in each system, there are factors of greater and lesser influence. The Pareto-Lorenz analysis is based upon the thesis of Vilfredo Pareto and Joseph Juran, according to which 80% of all qualitative problems are caused by 20% of the identified reasons for which it becomes likely to undertake compensatory and preventive actions. Pareto’s principle makes it possible to find 20% of the causes that bring about 80% of losses [25].

## 3. Results

The study included 312 respondents treated for osteoporosis, i.e., patients in osteoporosis clinics. The vast majority of the study participants were women who lived in a city. The greatest percentage of the study group participants had secondary education. The vast majority of respondents assessed their financial situation as good. In assessing current employment status within the study group, slightly more than half of all respondents were retired/pensioners. Over half of all respondents had been treated for osteoporosis for 1–5 years. Slightly more than half of the participants evaluated their own health condition as good (Table 1).

### 3.1. PASAT POZ Tool

When asked to provide a general assessment of the clinic they were attending, respondents most often indicated the answer “very good” (37.5%). Slightly less frequent responses included: “good” (29.2%) and “rather good” (26.3%), and only 7.1% of all study participants assessed their clinic as “bad”. For the significant majority of persons (92.9%), their presence at the clinic was in order to visit a specialist. Only 7.1% of all patients had planned diagnostic tests.

In the PASAT POZ tool, six dimensions for the assessment of clinics were analyzed. The result of each dimension was set on a scale from 1 to 5, where 1 means very bad and 5 very good. Taking into account the averages and medians calculated for all respondents, the professional activities of the nurses, the availability of information and the clinic itself were rated the highest (good). Medical services were evaluated slightly less favorably (between good and rather good) and the lowest (rather good) rating was assigned to the extent of information received from the doctor during the visit and the doctor’s professional activities (Table 2).

Frequency of visits to the doctor significantly differentiates the dimensions of the assessment of a clinic. Respondents who visited the clinic from four to six times a year provided significantly worse assessments of their clinic, the medical services they received and the doctor’s professional activities than did those who visited it more than nine times a year. Respondents visiting their clinic from seven to nine times a year assessed the professional activities of nurses significantly better than did patients who did so from four to six times a year. Availability of information was also significantly better assessed by persons who visited their clinic seven to nine times a year than it was by those who did so less than four times a year (Table 3).

Period of treatment of osteoporosis linearly correlates with general assessments of the clinic and some dimensions of assessment according to the applied PASAT POZ tool (assessment of nurses’ professional activities, assessment of the clinic, assessment of doctor’s professional activities). The longer the treatment for osteoporosis, the lower the overall assessment of the clinic, indicating a lower level of satisfaction in general, as well as lower assessments concerning the clinic and nurses’ work (Table 4).

Our analysis indicates that there is a relationship between the age of respondents and the general assessment of the clinic and individual dimensions. Accordingly, the higher the study participants’ age, the lower the general assessment of the clinic and of satisfaction in the following dimensions: assessment of the clinic, assessment of medical services and assessment of nurses’ professional activities (Table 5).

We observed a statistically significant difference between women and men with regard to the expressed opinions of the general assessment of the clinic. Our analysis showed that women better assessed clinics overall. It is worth noting that the majority of women assessed their clinics as “very good”, while the majority of men evaluated their clinics as “rather good”; the response “bad” was given by only a few women and men (Table 6).

According to the PASAT POZ questionnaire, education linearly correlates with general assessments of the clinic and some dimensions of assessment, i.e., the higher the level of education of respondents, the better the general assessment of their clinic and of nurses’ professional activities (Table 7).

Our work demonstrates that when the PASAT POZ tool was applied, there was a relationship between financial situation and general assessments of the clinic and individual dimensions. Specifically, the better the financial situation of the respondents, the higher the general assessment and the higher the evaluations in the following dimensions: assessment of the clinic, of medical services and of nurses’ professional activities, as well as of doctors (Table 8).

The Student’s *t*-test for independent groups showed that the place of residence does not influence the dimensions of the assessments highlighted through the application of the PASAT POZ tool. Also, no relationship was observed between place of residence and general assessment of the clinic (Mann Whitney U test: Z = −0.925, *p* = 0.355).

A multivariate analysis was performed using linear regression models. The analysis showed no statistically significant results.

### 3.2. Pareto-Lorenz Analysis

The application of the PASAT POZ tool enabled the recognition of the importance of each specific aspect by indicating the percentages of negative assessments. The answers ‘very bad’, ‘bad’ or ‘no’ (depending on multiple-choice answers for a given question) were considered negative. A detailed evaluation of the outcome of the assessments allowed us to recognize the importance of aspects such as operating hours, staff politeness, cleanliness of toilets and rooms, equipment and aesthetics, as well as the signage of doctors’ offices. Among the assessed issues, patients most frequently evaluated the signage of doctors’ offices and cleanliness of rooms as “very good” and “good”. A small percentage of respondents indicated “bad” or “very bad” for issues of care, while the majority negatively assessed the politeness of reception desk staff and the clinic’s operating hours (Table 9).

With regard to the quality of the medical services offered to the patients, the possibility of obtaining basic diagnostic tests was the best assessed, i.e., as “good” and “very good” by the vast majority of the respondents. Unfortunately, the remaining aspects of medical services were not equally satisfying for the study participants. The worst assessed was the possibility of arranging home visits. The possibility of nursing care provided at the patient’s home was assessed slightly better (Table 10).

Among the attributes of nursing care, the best assessed were the kindness of nurses and diligence in terms of the performed procedures. The worst assessment concerned talking in a way that is understandable to the patient. Respondents notably assessed nursing care more highly than doctor’s care (Table 11).

The assessment of the availability of information on patients’ rights in osteoporosis clinics was positively assessed by the vast majority of respondents, and only a small percentage of the study group had problems with obtaining such information. The worst assessed aspect in our detailed assessment of the availability of information concerned the dissemination of information about preventive programs. When it comes to the availability of information on the type and prices of services provided in the clinic, the vast majority of respondents were satisfied (Table 12).

A detailed assessment of doctors’ professional activities allowed us to assess the most significant issues concerning patient satisfaction with care. In the case of primary health care outpatient clinics and specialist clinics, it is the doctor who is the decision-maker in the provision of health services. Respondents best assessed the aspect of talking in a way that is understandable to the patient and the kindness of the doctor. The worst-rated component of an appointment is the amount of time devoted to the patient (Table 13).

The majority of patients declared that they had received a satisfactory range of information from the doctor during the visit regarding their health condition, disease, problem, and methods of treatment. The smallest percentage of study participants provided a satisfactory assessment of the information they received from their doctor on the available procedures in case of deterioration/lack of health improvement. This means that almost half of all patients declared that they had not been informed enough in this regard (Table 14).

**Figure 1:**
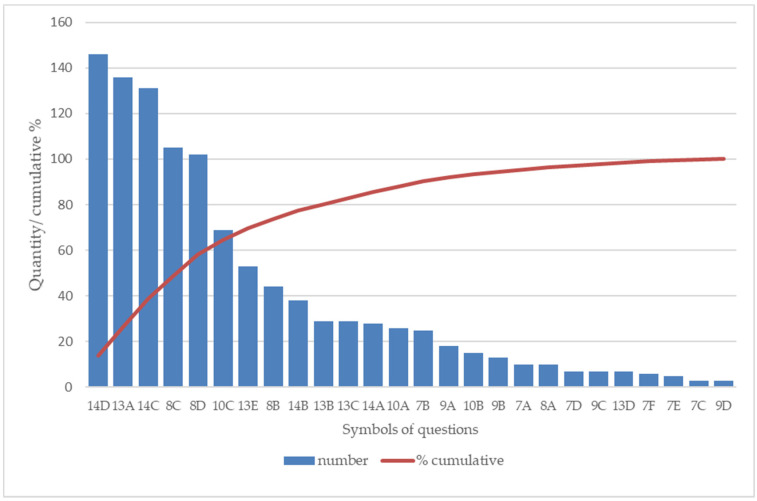
Pareto-Lorenz diagram for PASAT POZ tool.

Based on Table 8, Table 9, Table 10, Table 11, Table 12 and Table 13, a Pareto-Lorenz diagram (Figure 1) was created and Table 15 was constructed with the final results summarizing the Pareto-Lorenz analysis. The patients most often negatively assessed three aspects:

- range of information received from the doctor on the procedure in case of deterioration/lack of health improvement;
- amount of time devoted to the patient by the doctor;
- range of information received from the doctor on planned tests/procedures.

According to the assumptions of the Pareto-Lorenz analysis, the first two problems indicated by the study participants (i.e., lack of information received from their doctor on procedures in case of deterioration/lack of health improvement, limited time devoted to the patient by the doctor) accounted for over 20% of all existing problems (exactly 26.5%). Mitigation, therefore, of these issues is crucial for improving overall quality assessments of services. The addition of the third problem (i.e., little information received from the doctor about the planned tests/procedures) makes up 38.8% of all problems (low assessments) perceived by outpatients at osteoporosis clinics in this investigation.

## 4. Discussion

The performed analysis showed that the key element for the general assessment of the quality of services supplied by specialist osteoporosis clinic is the doctor. In this regard, patients most often negatively assessed three aspects: range of information received from the doctor on procedures in case of deterioration/lack of health improvement, time devoted to the patient by the doctor, and range of information received from the doctor on planned tests/procedures. Sociodemographic factors significantly determined degree of satisfaction with medical care. Our analysis showed that women better assessed clinics in overall terms. Additionally, the higher the study participant’s age, the lower the general assessment of the clinic. Our analysis showed that the better the financial situation of the respondents and the higher the level of education, the higher the general assessment of the clinic.

Patient satisfaction has become one of the most important measures of healthcare assessments and is increasingly used in the determination of the quality of care, partnerships between patients and healthcare providers and in the planning of health services [26,27]. Bleich, Özaltin and Murray hold that patient experience measures are very important in capturing the “responsiveness” of a healthcare system, and will ultimately lead to the definition of clearer priorities for quality improvements. The concept developed by the WHO is likely to gain even greater attention, as doctors and hospitals are under increasing pressure to improve the quality of care and patient safety while reducing the cost of medical services [28]. American researchers Dunsch et al. pointed to factors influencing satisfaction to the greatest extent, i.e., short waiting time for an appointment, clean rooms, and healthcare providers who respond to patients’ needs and treat them with respect [29]. Researchers Xesfing and Vozikis, who analyzed the impact of socioeconomic factors on satisfaction with care, indicated that the efficiency of health care systems translates directly into patient satisfaction [30], which may be the basis for the development of a satisfaction index for future health system assessments.

In our study, satisfaction with medical care was analyzed on the basis of the opinions of patients treated in osteoporosis clinics. We applied the PASAT POZ tool, and supplemented this with our own tool. One factor that influences overall satisfaction with medical care is waiting time for an appointment in the waiting room. A study by Med, Sci et al. in primary health care outpatient clinics in Riyadh indicated that a long waiting period, especially between registration and medical consultation, results in a higher percentage of dissatisfied patients [31]. This was confirmed by studies by Al-Harajin et al., carried out in Saudi Arabia, describing the results of patient satisfaction, which significantly differ depending on the waiting time for an appointment. Here, over 90% of all dissatisfied patients waited longer than 20 min between arrival, registration and medical consultation (*p* < 0.01) [32]. Unfortunately, studies indicate that the waiting time for an appointment in the Polish health care system is longer, e.g., research by Plentara et al., carried out in primary healthcare entities in the West Pomeranian Voivodeship, where 38% of all respondents noted a waiting time of more than 30 min [33].

In patient satisfaction studies, the subject of assessment is most often a medical appointment, which is the basis for the health system. Interdisciplinary research by Leźnicka et al. [34], conducted with the help of the PASAT toolkit in the Kuyavian-Pomeranian Voivodeship, showed that almost half (49%) of 2280 patients assessed the attending physicians very well when it came to listening carefully to the patient. In contrast, in our study, the same aspect was assessed as very good only by 22.1% and as good by 34.3% of all respondents. Ensuring intimacy by a doctor in the study by Leźnicka et al. was assessed very well by 45%, while in our study, this was the case for only 22.1%. The doctor talking in a way that is understandable to the patient in the study in the Kuyavian-Pomeranian Voivodeship was rated the highest by 45% of the respondents, and in our own study by 27.9%. It is also worth pointing out that in the study by Leźnicka et al., the amount of time devoted to the patient by the doctor was assessed as bad and very bad by only 6.8% of respondents, while in our study, negative assessments (bad and very bad) were indicated by as many as 43.6% of respondents, which may be an indication for health care units to take corrective actions [34].

As seen from such comparisons among studies, patients differ in their preferences as to the hierarchy of importance of medical care aspects; nonetheless, professionalism and the availability of a doctor comprise core factors. This was confirmed by the 2014 and 2015 study of satisfaction with care conducted by Faye et al. from Columbia University in New York on people with celiac disease. In that study, respondents declared significantly higher satisfaction if they felt that their doctor or dietitian was easily accessible if necessary (87%, *p* < 0.001) [35]. Other researchers, including Kotzian, Kutney Lee and McHugh, concluded that a relatively low percentage of doctors per capita may significantly reduce satisfaction rates [36,37]. Patient satisfaction with nursing care in our own study was assessed on a higher level than that with doctor’s care. On the basis of a satisfaction survey, Przychodzka et al. indicated areas of nursing care that were poorly rated by patients, i.e., related to aspects including information provided by the nurse and the amount of time that nurses were available to talk with them [38]. In our study, the lowest evaluation of nurses’ work, in the opinion of patients of osteoporosis clinics, concerned talking in a way that patient could understand.

The Pareto-Lorenz analysis allowed us to identify the most relevant negative factors in the functioning of a medical facility. It is a tool that can be applied to improve quality, as its use enables the elimination of false flags and indicates ways of enhancing current activities, and, consequently, increasing efficiency [25]. The Pareto analysis of data in a study by Gupta et al. on the satisfaction of patients from nine district hospitals in Bihar indicated that increasing patient satisfaction by more than 60% can be achieved by referring to the three highest attributes of dissatisfaction, i.e., a lack of availability of medicines, unsatisfactory time of consultation and cleanliness of the rooms [39]. The Pareto analysis of the research conducted by the Leźnicka et al., carried out with the help of the PASAT toolkit on 2280 patients, indicated that as many as one in four respondents (25%) declared a problem with information on patients’ rights. In our study, two aspects accounted for 26.5% of all problems: little information received from a doctor on procedures in case of deterioration/lack of health improvement, and limited amount of time devoted to the patient by the physician. Respondents in our study expected more attention from the doctor and the extension of the information process. Ghose and Adhish observed that patient satisfaction was largely influenced by the time devoted to the patient and by the ordering of tests, and a high percentage of patients were satisfied with the services provided by their doctor in terms of attributes such as doctor availability, medical care and treatment received [40].

Many researchers have attempted to determine the relationship between sociodemographic data and satisfaction with care. Our study showed that there is a relationship between age and the level of satisfaction with care, i.e., the higher the age, the lower the general assessment of the clinic (*p* < 0.05). An example of different dependencies is evident in Uzun [41], in which patients over 65 provided significantly better assessments of quality of care than did patients under the age of 65.

The health care sector is a specific area of activity; it is highly sensitive to quality issues, because where human life and health are concerned, high-quality services should be available and considered a right [42]. Our study is the first to show the degrees of satisfaction with medical care of patients treated for a chronic disease based on the use of a validated tool. The present study revealed the strengths and weaknesses of patient care and the functioning of the clinics.

A literature review provided information on a large number of studies on patient satisfaction with medical services. However, the analyses presented by researchers were often not unified and were carried out with the use of non-validated tools, which is why it is difficult to compare them reliably. In this work, the indicators of the quality of medical care and satisfaction with medical services of people suffering from osteoporosis were also not analyzed. Foreign researchers have undertaken analyses of satisfaction with osteoporosis treatment, but these studies covered issues related to treatment regimens, adherence to medical recommendations and comparisons of effects in the treatment of osteoporosis, rather than patients’ feelings about the care they had received.

This study ensured the anonymity of the respondents. Access to patients’ medical documentation would allow more detailed analyses to be made of other medical aspects and their possible impact on satisfaction with medical care. These limitations warrant further investigations with regard to satisfaction of medical care and chronic disease such as osteoporosis. Furthermore, more detailed analyses of the treatment process would enable better assessments of quality of care.

### Future Directions

It is advisable for health service providers to regularly monitor the quality of the health care they offer and make improvements in order to increase patient satisfaction rates. Therefore, further research is needed, especially in relation to chronic diseases such as osteoporosis. Research showing the subjective feelings of patients regarding the medical procedures performed may be a hint for management staff regarding the expectations of patients, and may indicate possible directions for the implementation of new solutions.

## 5. Conclusions

The level of satisfaction of patients treated in osteoporosis clinics with medical care is different for the assessment of doctors and nurses. Patients assessed the work of the nurses the best, while that of the doctor, who was most often associated with negative aspects of care, was evaluated significantly worse. The results indicate that the duration of osteoporosis treatment is a factor that significantly influences satisfaction with medical care, i.e., the longer the treatment time for osteoporosis, the lower the clinic’s assessment, and therefore, the lower the patient satisfaction. Most sociodemographic factors were found to be significantly related to the examined aspects among the respondents.

## Figures and Tables

**Table 1:** Socio-demographic characteristics of the study population.

Variable	Characteristic	Frequency (N = 312)	%
**Sex**	Male	26	8.3%
	Female	286	91.7%
**Place of residence**	City	192	61.5%
	Rural areas	120	38.5%
**Educational status**	Higher education	69	22.1%
	Secondary education	127	40.7%
	Vocational education	86	27.6%
	Primary education	30	9.6%
**Financial situation**	Very good	37	11.9%
	Good	223	71.5%
	Bad	49	15.7%
	Very bad	3	1%
**Employment status**	Retired and pensioners	175	56.1%
	White-collar worker	70	22.4%
	Manual workers	51	16.3%
	Unemployed	16	5.1%
**Duration of the osteoporosis treatment**	1–5 years	196	62.9%
	6–10 years	86	27.6%
	>10 years	30	9.6%
**Self-assessment of health condition**	Very good	12	3.8%
	Good	176	56.4%
	bad	120	38.5%
	Very bad	4	1.3%

**Table 2:** Descriptive statistics of the dimensions of clinic assessments using the PASAT POZ tool.

PASAT POZ	Min	Max	M	Me	SD	IQR
Assessment of the clinic	2.67	5.00	4.02	4.00	0.64	1.0
Assessment of medical services	2.00	5.00	3.52	3.50	0.76	1.0
Assessment of nurses’ professional activities	2.50	5.00	4.18	4.13	0.65	1.0
Assessment of information availability	1.00	5.00	4.18	4.33	0.85	1.0
Assessment of doctor’s professional activities	2.00	3.80	3.01	3.00	0.35	1.2
Assessment of the extent of information received from the doctor during the visit	1.25	4.50	3.12	3.00	0.57	1.0

Abbreviations: Min, minimum; Max, maximum; M, mean; Me, median; SD, standard deviation; interquartile range, IQR.

**Table 3:** Number of visits per year and assessment of individual dimensions of the clinic’s operation.

PASAT POZ	How Many Times in the Last Year Did You See a Doctor or Have Doctor Home Appointment?								ANOVA		Turkey’s Test
	<4 (1)		4–6 (2)		7–9 (3)		>9 (4)				
	M	SD	M	SD	M	SD	M	SD	F	*p*	
**Assessment of the clinic**	4.05	0.65	3.87	0.67	4.07	0.58	4.22	0.64	3.50	0.016 *	2/4
Assessment of medical services	3.50	0.78	3.38	0.72	3.55	0.76	3.82	0.78	3.21	0.023 *	2/4
Assessment of nurses’ professional activities	4.14	0.64	4.03	0.68	4.28	0.60	4.30	0.73	3.22	0.023 *	2/3
Assessment of information availability	3.95	0.98	4.23	0.77	4.31	0.77	3.95	1.01	3.21	0.023 *	1/3
Assessment of doctor’s professional activities	3.04	043	2.95	0.33	3.00	0.33	3.15	0.34	3.14	0.025 *	2/4
Assessment of the extent of information received from the doctor during the visit	3.18	0.50	3.08	0.61	3.14	0.59	3.07	0.53	0.50	0.682	ns

* statistically significant. Abbreviations: M, mean; SD, standard deviation; ns, non-significant.

**Table 4:** Correlations between the period of osteoporosis treatment and dimensions and overall assessment of the clinic.

PASAT POZ	Period of Osteoporosis Treatment	
	rho	*p*
Assessment of the clinic	−0.162	0.004 *
Assessment of medical services	−0.062	0.272
Assessment of nurses’ professional activities	−0.197	0.000 *
Assessment of information availability	−0.022	0.699
Assessment of doctor’s professional activities	−0.155	0.036 *
Assessment of the extent of information received from the doctor during the visit	0.045	0.431
General assessment of the clinic	−0.205	0.000 *

* statistically significant.

**Table 5:** Correlations between age and dimensions and general assessment of the clinic.

PASAT POZ	Age in Years	
	R/rho	*p*
Assessment of the clinic	−0.199	0.000 *
Assessment of medical services	−0.113	0.045 *
Assessment of nurses’ professional activities	−0.162	0.004 *
Assessment of information availability	−0.069	0.222
Assessment of doctor’s professional activities	−0.067	0.235
Assessment of the extent of information received from the doctor during the visit	0.007	0.901
General assessment of the clinic	−0.200	0.003 *

* statistically significant.

**Table 6:** Sex and a general assessment of the clinic.

How Do You Generally Evaluate the Clinic?	Sex				Total	
	Woman		Man			
	N	%	N	%	N	%
Very good	110	38.5%	7	26.9%	117	37.5%
Good	84	29.4%	7	26.9%	91	29.2%
Rather good	72	25.2%	10	38.5%	82	26.3%
Bad	20	7.0%	2	7.7%	22	7.1%
In total	286	100.0%	26	100.0%	312	100.0%
Mann Whitney U test: Z = −1.392. *p* = 0.048 *						

* statistically significant. Abbreviations: N, number.

**Table 7:** Correlations between education and dimensions and general assessment of the clinic.

PASAT POZ	Education	
	rho	*p*
Assessment of the clinic	0.160	0.005 *
Assessment of provided medical services	0.099	0.081
Assessment of nurses’ professional activities	0.146	0.010 *
Assessment of information availability	0.050	0.381
Assessment of doctor’s professional activities	0.072	0.206
Assessment of the extent of information received from the doctor during the visit	0.045	0.433
General assessment of the clinic	0.119	0.035 *

* statistically significant.

**Table 8:** Correlations between financial situation and dimensions and general assessment of the clinic.

PASAT POZ	Financial Situation	
	rho	*p*
Assessment of the clinic	0.257	0.000 *
Assessment of medical services	0.170	0.003 *
Assessment of nurses’ professional activities	0.170	0.003 *
Assessment of information availability	0.024	0.673
Assessment of doctor’s professional activities	0.161	0.004 *
Assessment of the extent of information received from the doctor during the visit	0.029	0.615
General assessment of the clinic	0.229	0.000 *

* statistically significant.

**Table 9:** Detailed assessment of the clinics.

Assessment of the Clinic	Very Good		Good		Rather Good		Bad		Very Bad		Total	
	N	%	N	%	N	%	N	%	N	%	N	%
Clinic’s operating hours	79	25.3	140	44.9	83	26.6	10	3.2	0	0	312	100.0
Politeness of reception desk staff	96	30.8	129	41.3	62	19.9	23	7.4	2	0.6	312	100.0
Cleanliness of rooms	86	27.6	162	51.9	61	19.6	3	1	0	0	312	100.0
Equipment and aesthetics of the rooms	83	26.6	154	49.4	68	21.8	7	2.2	0	0	312	100.0
Cleanliness of toilets	87	27.9	158	50.6	62	19.9	5	1.6	0	0	312	100.0
Signage of doctors’ offices	99	31.7	162	51.9	45	14.4	6	1.9	0	0	312	100.0

Abbreviations: N, number.

**Table 10:** Detailed assessment of medical services.

Assessment of Medical Services	Very Good		Good		Rather Good		Bad		Very Bad		Total	
	N	%	N	%	N	%	N	%	N	%	N	%
Possibility of performing basic diagnostic tests	97	31.1	135	43.3	70	22.4	10	3.2	0	0.0	312	100.0
Possibility of performing specialist diagnostic tests	80	25.6	101	32.4	87	27.9	44	14.1	0	0.0	312	100.0
Possibility of arranging home visits	38	12.2	80	25.6	89	28.5	103	33.0	2	0.6	312	100.0
Possibility of nursing care provided at patient’s home	28	9.0	108	34.6	73	23.4	100	32.1	2	0.6	312	100.0

Abbreviations: N, number.

**Table 11:** Detailed assessment of nurses’ professional activities.

Assessment of Nurses’ Professional Activities	Very Good		Good		Rather Good		Bad		Total	
	N	%	N	%	N	%	N	%	N	%
Kindness of nurses	142	45.5	124	39.7	28	9.0	18	5.8	312	100.0
Talking in a way that is understandable to the patient	110	35.3	137	43.9	52	16.7	13	4.2	312	100.0
Diligence of performed procedures	125	40.1	136	43.6	44	14.1	7	2.2	312	100.0
Ensuring intimacy/privacy	109	34.9	144	46.2	56	17.9	3	1.0	312	100.0

Abbreviations: N, number.

**Table 12:** Detailed assessment of the availability of information.

Detailed Assessment of the Availability of Information on	Yes		Rather Yes		No		I Do Not Need		Total	
	N	%	N	%	N	%	N	%	N	%
Type and prices of services provided in the clinic	204	65.4	44	14.1	26	8.3	38	12.2	312	100.0
Patients’ rights	237	76.0	52	16.7	15	4.8	8	2.6	312	100.0
Preventive programs	220	70.5	23	7.4	69	22.1	0	0.0	312	100.0

Abbreviations: N, number.

**Table 13:** Detailed assessment of doctor’s professional activities.

Assessment of Doctor’s Professional Activities	Very Good		Good		Rather Good		Bad		Very Bad		Total	
	N	%	N	%	N	%	N	%	N	%	N	%
Amount of time devoted to the patient	64	20.5	51	16.3	61	19.6	136	43.6	0	0.0	312	100.0
Listening carefully to the patient	69	22.1	107	34.3	107	34.3	29	9.3	0	0.0	312	100.0
Ensuring intimacy/privacy	69	22.1	107	34.3	107	34.3	29	9.3	0	0.0	312	100.0
Talking in a way that is understandable to the patient	87	27.9	121	38.8	97	31.1	7	2.2	0	0.0	312	100.0
Kindness of the doctor	82	26.3	103	33.0	74	23.7	52	16.7	1	0.3	312	100.0

Abbreviations: N, number.

**Table 14:** Detailed assessment of the range of information received from the doctor during the visit.

Assessment of Range of Information Received from the Doctor during the Visit on	Yes		Rather Yes		No		I Don’t Need		Total	
	N	%	N	%	N	%	N	%	N	%
Health condition, disease, problem which he/she reported	206	66.0	75	24.0	28	9.0	3	1.0	312	100.0
Methods of treatment	187	59.9	87	27.9	38	12.2	0	0.0	312	100.0
Planned tests/procedures	103	33.0	72	23.1	131	42.0	6	1.9	312	100.0
Procedure in case of deterioration/lack of health improvement	88	28.2	68	21.8	146	46.8	10	3.2	312	100.0

Abbreviations: N, number.

**Table 15:** The frequency of difficulties (negative assessments) regarding detailed aspects of the quality of medical services provided by clinics.

Aspect	N	% of Observations	% of All
Assessment of range of information received from the doctor on procedures in case of deterioration/lack of health improvement	146	46.8%	13.7%
Amount of time devoted to the patient by the doctor	136	43.6%	12.8%
Assessment of range of information received from the doctor on planned tests/procedures	131	42.0%	12.3%
Possibility of arranging home visits	105	33.7%	9.9%
Possibility of nursing care provided at patient’s home	102	32.7%	9.6%
Assessment of the availability of information on preventive programs	69	22.1%	6.5%
Kindness of the doctor	53	17.0%	5.0%
Possibility of performing specialist diagnostic tests	44	14.1%	4.1%
Assessment of range of information received from the doctor on methods of treatment	38	12.2%	3.6%
Doctor’s listening skills	29	9.3%	2.7%
Ensuring intimacy/privacy by the doctor	29	9.3%	2.7%
Assessment of range of information received from the doctor on health condition, disease or reported problem	28	9.0%	2.6%
Assessment of the availability of information on type and prices of services provided in the clinic	26	8.3%	2.4%
Politeness of reception desk staff	25	8.0%	2.3%
Kindness of nurses	18	5.8%	1.7%
Assessment of the availability of information on patient’s rights	15	4.8%	1.4%
The nurses’ ability to talk in a way that is understandable by the patient	13	4.2%	1.2%
Operating hours of the clinic	10	3.2%	0.9%
Possibility of performing basic diagnostic tests	10	3.2%	0.9%
Equipment and aesthetics of the rooms	7	2.2%	0.7%
Diligence of performed procedures by nurses	7	2.2%	0.7%
The doctor’s ability to talk in a way that is understandable by the patient	7	2.2%	0.7%
Signage of doctors’ offices	6	1.9%	0.6%
Cleanliness of toilets	5	1.6%	0.5%
Cleanliness of rooms	3	1.0%	0.3%
Ensuring intimacy/privacy by nurses	3	1.0%	0.3%

Abbreviations: N, number.

## Data Availability

Not applicable.
